# Clinical Application of Antibacterial Hydrogel and Coating in Orthopaedic and Traumatology Surgery

**DOI:** 10.3390/gels7030126

**Published:** 2021-08-25

**Authors:** Daniele De Meo, Giancarlo Ceccarelli, Giancarlo Iaiani, Federico Lo Torto, Diego Ribuffo, Pietro Persiani, Ciro Villani

**Affiliations:** 1 Orthopaedic and Traumatology Unit, Department of General Surgery, Plastic Surgery, Orthopedics, Policlinico Umberto I Hospital—Sapienza, University of Rome, Piazzale A. Moro 3, 00185 Rome, Italy; ppersiani@me.com (P.P.); ciro.villani@uniroma1.it (C.V.); 2 M.I.T.O. (Infections in Traumatology and Orthopedics Surgery) Study Group, Policlinico Umberto I Hospital, Viale del Policlinico 155, 00161 Rome, Italy; giancarlo.ceccarelli@uniroma1.it (G.C.); giancarlo.iaiani@uniroma1.it (G.I.); federico.lotorto@uniroma1.it (F.L.T.); diego.ribuffo@uniroma1.it (D.R.); 3 Department of Public Health and Infectious Diseases—Sapienza, University of Rome, Piazzale A. Moro 5, 00185 Rome, Italy; 4 Plastic Surgery Unit, Department of General Surgery, Plastic Surgery, Orthopedics, Policlinico Umberto I Hospital—Sapienza University of Rome, Viale del Policlinico 155, 00161 Rome, Italy

**Keywords:** periprosthetic joint infection, fracture related infection, osteomyelitis, coating, antibacterial hydrogel, open fractures, infection, osteosynthesis, gentamicin-coated nail, antibiotic-coated nail

## Abstract

Implant related infection is one of the most frequent complications in orthopaedic and trauma surgery. Local antibiotic treatment strategies are becoming part of the prevention and treatment methodology for this fearful complication. To date, there are two coatings available on the market, both with a polylactic acid base. Current evidence supports the use of these types of coatings in the prophylaxis of periprosthetic infections and fracture-related infections. However, their therapeutic use has been less investigated. The purpose of this article is to summarise recent evidence relating to the clinical application of antibacterial hydrogels and coatings in orthopaedic and traumatology surgery and indicating which future applications may benefit from it.

## 1. Introduction

Implant-related infection is one of the most frequent complications in orthopaedic and trauma surgery. It represents a great challenge for the orthopaedic surgeon and forces the patient to undergo frequent multiple surgical treatments, resulting in a functional deficit for said patient and an increase in expenditure for the national health systems.

Periprosthetic joint infections (PJIs) have an increasing incidence that currently stands at around 1.3% [1], resulting in the third or fourth cause of revision at 8.8–18.6% [2,3]. In surgical re-operations it represents 22% of the revision causes [4].

Fracture-related infections (FRIs) can have an even higher incidence, reaching 6–33% in certain districts (i.e., tibia) [5]. Such infections can lead to an increase in hospitalization costs, of up to seven times the hospitalization costs of a non-infected patient [6]. The need to develop incidence reduction strategies and reduce related costs has led to the development of strategies for various local carrier and coating systems. In recent years, researchers have placed a great deal of interest in recent years in the development of hydrogels that can be applied in medicine and surgery [7,8]. Among these, the use of antibiotic coatings and hydrogels are among the few strategies currently available on the market. These coatings work by preventing bacterial adhesion on the surface of the implants, thus preventing the formation of biofilms.

There are two coating systems currently available on the market. The first is a biocompatible hydrogel, the Defensive Antibacterial Coating (DAC—Novagenit, Mezzolombardo, Italy) composed by covalently bound hyaluronic acid and polylactic acid capable of forming a physical barrier against bacterial adhesion by releasing high concentrations of the surgeon’s chosen antibiotic or antifungal [9,10]. It subsequently undergoes complete reabsorption via hydrolytic degradation within 72 h, completely releasing the antibiotic contained within it. In vitro studies have shown that bacterial colonization is significantly reduced if the hydrophobic surface of the implant is changed to hydrophilic and the hyaluronan coating allows that to happen. In addition, hyaluronan also possesses bacteriostatic effects. The possibility of using different antibiotics on different implants makes it extremely versatile. Its safety and efficacy profile has been tested both in vitro and in vivo [11,12]. Manufacturers recommend their use in the prevention of infections.

The second system is an antibiotic coating available only for ETN PROtect intramedullary tibial nailing (DePuy Synthes Companies, Zuchwil, Switzerland). The coating, which is applied directly in the production phase and then delivered to the surgeon already coated, consists of an absorbable poly (d, l-lactide) (PDLLA) matrix with gentamicin sulphate incorporated [13,14]. The application of gentamicin guarantees an estimated release of 80% within 48 h following implantation, combining a massive initial release associated with a secondary timed release: this by virtue of the initial break down of the most superficial layer of the matrix and the subsequent break down of the exposed polymer [15]. Its safety has been studied, as the local release of high doses of gentamicin does not generate levels of systemic toxicity [16]. The manufacturers recommend the use of the implant for the prevention of infections in high-risk patients (polytrauma, open fractures, immuno-suppressed subjects) and in infection related revision surgeries.

The main features of both coatings are shown in Table 1. Both coating systems have shown good clinical efficacy in the clinical studies reported thus far. The purpose of the study is to summarize recent evidence relating to the clinical application of antibacterial hydrogel and coating in orthopaedic and traumatology surgery, indicating which future prospects could benefit from its applications.

## 2. Periprosthetic Joint Infections

### 2.1. Prophylactic Use in Joint Replacement

**Figure 1:**
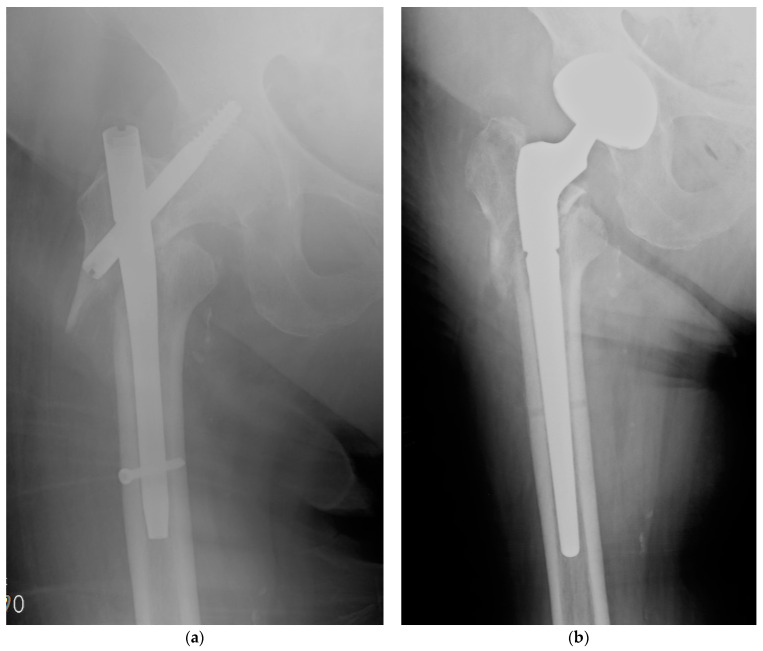
Clinical case #1: woman, 89 years old, hip fracture osteosynthesis failure at around 2 months post trauma (**a**). Removal of intramedullary nail and subsequent total hip replacement with implantation of a revision stem coated with 10 mL DAC gel with gentamicin and vancomycin (**b**).

The use of antibacterial coating in prosthetic surgery was initially validated by a European multicentric study [17]. Romanò et al. showed a significant reduction in the incidence of periprosthetic infections in the treatment group compared to the control group (0.6% vs. 6%; *p* = 0.003). The use of this type of coating is analogous—in uncemented prostheses—to the antibiotic loaded bone cement in cemented prostheses. Not surprisingly, the antibiotics used in the study were the same as those added to the cement in cemented prostheses (gentamicin and vancomycin). The application of the gel does not affect the osseointegration of the prosthetic implant. This study was later supported by other evidence in the literature that confirm its effectiveness. A previous study shows that, in patients who underwent a cementless revision due to aseptic failure of the hip replacement, the incidence of infection was the main cause of short-term failure and that the application of the gel drastically reduces this occurrence (*p* = 0.0001) [18]. In cases of uncemented implants, the use of the gel in revisions and re-interventions represents an important reduction in the infectious risk (Figure 1).

With regard to mega-prostheses, Zoccali et al. showed a reduction in the risk of infection, similar to that in cases of prosthetic revision, with no evidence of infections in the treatment group at the 2-year follow-up [19].

Although a routine use for all patients has not yet been put forth, a cost-benefit study proposed by Trentinaglia et al. simulated how the routine use of antibiotic gel on all non-cemented prosthetic surgery implants, similar to the use of antibiotic-loaded cement in cemented prostheses, would reduce the management costs of infected patients, with greater savings compared to other types of surface coatings, such as the silver-coating [20]. Recently, with regard to high-risk patients, there have also been proposals of risk reduction strategies using calcium sulphate antibiotic-loaded beads (Stimulan; Biocomposites Ltd., Keele, UK) with the same antibiotics [21]. However, the application of this type of local antibiotic carrier is burdened by a variable risk of complications such as hypercalcemia, heterotopic ossifications and prolonged secretion from the surgical wound, which appear to be closely related to the application site and the quantity of the beads used [22]. Therefore, the authors believe that the application of antibiotic hydrogel is equally as effective without an increase of short and medium-term risks, despite its reabsorption (and therefore the local release of antibiotic) occurring in a faster manner compared to the calcium sulphate antibiotic-loaded beads, that in fact is capable of maintaining high local antibiotic concentrations for more than a month. Although there are no studies that directly compare its long-term efficacy, the release within the first post-operative hours guaranteed by the hydrogel appears to be sufficient to protect the implant from the formation of biofilm.

On the basis of the aforementioned evidence currently available in the literature, the authors recommend the use of antibiotic coatings in cases at increased risk of infection: revisions, mega-prostheses, but also patients with known systemic risks such as rheumatological diseases, diabetes, previous bone infections in other districts, and prostheses resulting from fracture or failure of the means of synthesis. Larger prospective multicentric studies are needed to establish whether a routine application to all prostheses implantations is indicated.

### 2.2. Therapeutic Use in PJI

The application of hydrogel as part of a therapeutic strategy has been less investigated than its prophylactic efficacy and there are only case series that study its application. In these cases, the gel acts both as a local delivery system of a targeted antibiotic therapy and as protection to the newly implanted device, in a one or two stage exchange (OSE and TSE; in general, OSE is a procedure in which the infected prosthesis is replaced directly with a new one, TSE involves removal of the prosthesis and placement of an antibiotic spacer in a first surgery, followed by removal of the spacer and re-implantation of the prosthesis in a second one). In particular, its application can be useful when the revision strategy involves the implantation of a cementless revision prosthesis, in cases, that is, where bone cement cannot be used as a local antibiotic delivery system and the prosthetic interface in contact with the bone is left without targeted protection against the pathogen responsible for a PJI. In 2018, Zagra et al. tested the effectiveness of the gel in TSE compared to a control group (0 vs. 14.8% of infections, *p* = 0.11), observing a significant difference in terms of hospital stay (*p* < 0.0001) [23].

In the same year, a non-inferiority study compared the use of gel in the OSE to the TSE without gel, observing similar relapse rates, with longer hospitalisation times and duration of systemic antibiotic therapy in the TSE group [24]. Recently, a further study tested the application of the antibiotic gel as part of an OSE strategy in a group of 10 patients with a minimum follow-up of 2 years, resulting in no failures [25].

**Figure 2:**
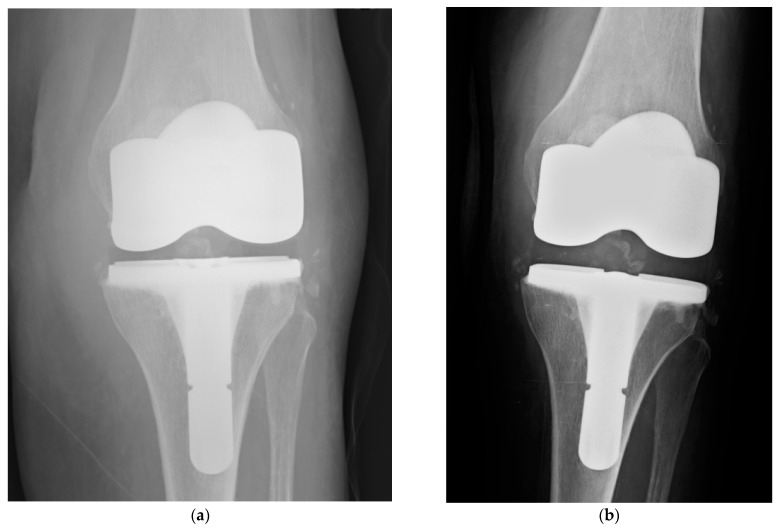
Clinical case #2: woman, 82 years old. Late acute haematogenous knee PJI from *Escherichia coli* (**a**) treated with DAIR plus DAC gel 10 mL (gentamicin + meropenem), followed by 12 weeks of targeted antibiotic therapy. Check-up at 18 months with no signs of infection (**b**).

To date, there are no studies describing the use of antibiotic gel in debridement, antibiotic and implant retention procedures (DAIR) for acute peri-prosthetic infections. In our clinical practice, with regard to the DAIR surgical treatment protocol—along with the replacement of the mobile components, the irrigation with at least 9 L of physiological solution and antiseptic solutions (povidone iodine and hydrogen peroxide), the packaging of a new surgical field after the first debridement—the antibiotic gel is applied with the choice of antibiotic selected on the basis of the antibiogram, if available, before closing the joint capsule (Figure 2). However, in our opinion, there is a need for randomised clinical trials (RCTs) to rigorously explore its efficacy.

There is, on the other hand, literature regarding the efficacy of calcium sulphate antibiotic-loaded beads used as a local delivery system, which do not act as a coating, but are, however, capable of a longer-lasting targeted local antibiotic release [22]. This carrier was also tested in the DAIR strategy, modified in “debridement, antibiotic pearls, and retention of the implant, DAPRI”, where the use of the beads is associated with a debridement strategy associated with the use of an argon beam for the dislodgment of the biofilm from the implant in 10 patients, with promising results [26].

The use of hydrogels as a carrier has also been tested in cases of extreme rescue, where other strategies were needed due to the impossibility of resorting to traditional techniques. Recently, the Lion NJI Study Group experimented the innovative potential of hydrogels as a local delivery system of bacteriophages. In this study, Ferry et al. investigated the anti-biofilm activity of bacteriophages incorporated in the hydrogel, demonstrating their bactericidal capacity in vitro and presenting a clinical case of rescue, which failed, though not due to a recurrence of the *Staphylococcus aureus* strain infection against which the pathogen was directed [27]. The combination of therapy with phages and the use of the gel as their vector opens up new therapeutic frontiers that must essentially be monitored, given the growing increase in antibiotic resistance.

## 3. Fracture Related Infections

### 3.1. Prophylactic Use in Fracture Fixations

The efficacy and safety of the DAC in this field were tested via a multicentric study [28]. In this RCT, the authors compared 126 cases treated with hydrogel supplemented with gentamicin, vancomycin and meropenem to a control group of 127 patients, reporting 0 surgical site infections in the treated cases and 6 in the control group (*p* = 0.03). The application of the gel did not affect the formation of the bone callus.

In tibial fractures, which are burdened by an increased risk of infection compared to other districts due to their more frequent exposure and trauma of the surrounding soft tissues, the use of an antibiotic coating based on gentamicin has proven to be an effective strategy in patients at high risk of infection. A recent systematic review studied all the cases in which the nail was used: of the 105 patients available for follow-up, 4 developed an FRI (3.81%) and 3 a superficial surgical site infection (2.86%) [29]. However, the data deriving from this systematic review were predominantly derived from case series; in the only prospective randomized study available regarding the use of nails with antibiotic coating, Pinto et al. showed a significant reduction in the incidence of FRIs compared to the controls (*p* = 0.031) [30]. On the basis of the available data, the use of the pre-loaded PDLLA-antibiotic coatings has the advantage of having a prompt application of the product directly on the implant that, however, it is limited to the combination with a single type of antibiotic and limited to a single type of intramedullary implant—future studies are needed to test this type of implant on a larger scale and on different tibial nail implants.

**Figure 3:**
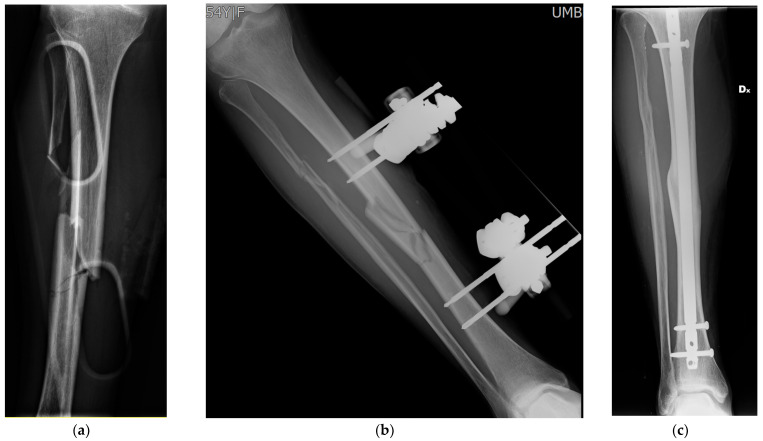
Clinical case #3: woman, 55 years old. Open right (“Dx” in the image) tibia and fibula fracture (Gustilo 3 A) (**a**) treated with temporary stabilisation with an external fixator (**b**), synthesised with ENT PROtect intramedullary nail coated with gentamicin and DAC gel with vancomycin on the 25th day. Check-up at 18 months with fracture healing and no signs of infection (**c**).

In our experience, based on the aforementioned evidence, the application of antibiotic coatings is recommended in cases of open fractures, in the definitive internal fixation treatment (Figure 3).

Its use can also be recommended in cases where the patient has clinical characteristics of high risk of infection: diabetic patients, smokers, morbidly obese and those with poor soft tissue envelopes. The use of either vancomycin, gentamicin, or both, is re-modulated on the basis of available antibiotic bone cement experiences.

### 3.2. Therapeutic Use in FRI

There is very little literature regarding the therapeutic use of gels in FRI. The available reports envisage the use of these antibiotic coatings as part of a two-stage strategy based on a first resection time and a subsequent restorative time, in which the coating protects the final implant [29]. In a case series, Metsemakers et al. used gentamicin PDLLA coated nails as part of the reconstructive strategy in 4 patients, reporting bone union in 3 out of 4 cases [31]. Mogghadam et al. analysed 33 patients with tibial non-union, of which 22 were infected, in which the nail was applied in the second part of the Masquelet technique by adding reamer-irrigator-aspirator (RIA), material from the ipsi- or contralateral femur, bone morphogenetic protein (BMP-) 7 and tricalcium phosphate to fill the bone defect [32]. Bone healing was achieved in 80.6% of cases.

With regard to the hydrogel DAC, the only study dealing with post-traumatic bone infections is the study by Corona et al., in which they evaluated the effectiveness of the gel in protecting the mega-prostheses implanted to fill the infected post-traumatic bone defects. [33]. At a minimum follow-up of 1 year, none of the 11 patients who underwent mega-prosthesis implantation developed infection; implant survival at 4 years was 81.8%.

**Figure 4:**
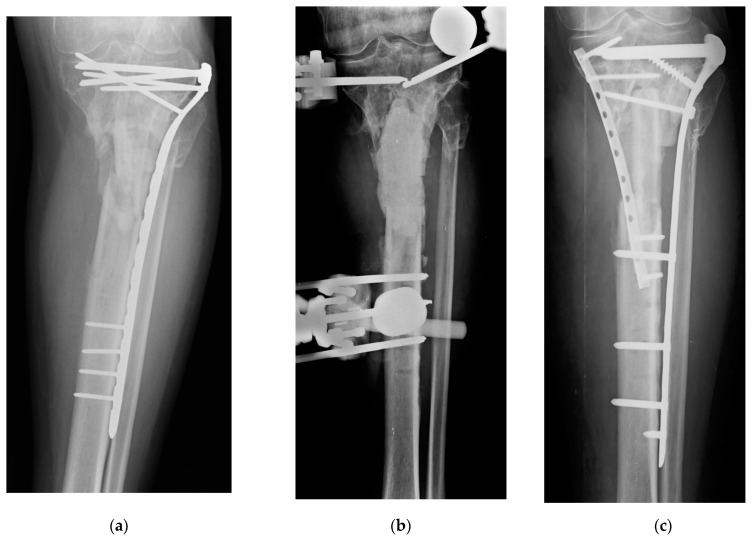
Clinical case #4: man, 44 years old. FRI of the proximal tibia following a car accident polytrauma (**a**), treated with a biological chamber technique by first removing synthesis media and infected tissue, positioning the antibiotic loaded bone cement with gentamicin and vancomycin, stabilising with an external fixator (**b**) and subsequently reconstructing with contralateral fibula grafting, RIA bone grafting, osteosynthesis with plate and screws coated with 10 mL of DAC gel with vancomycin and gentamicin. Check-up at 4 months without clinical and laboratory signs of infection and initial signs of infection site healing (**c**).

From the available data, the application of the antibiotic gel appears to be a promising strategy for the protection of the new implants also in cases of FRI, however they need to be confirmed with prospective studies containing a wider range of cases. In the experience of the authors, the application of the gel on the plate and screws positioned during the reconstructive surgery of the Masquelet technique allows an additional protection, directed at the pathogen isolated during the first surgical stage, though correct management of the perioperative antibiotic therapy is irreplaceable (Figure 4).

## 4. Conclusions

The clinical application of antibiotic gels and coatings is becoming more and more prominent within the prophylaxis and multidisciplinary treatment of implant-related infections. Its clinical results are promising though based mainly on low-number retrospective studies, especially in the area of fracture-related infections. Prospective randomized studies are needed in order to determine their effectiveness in different situations.

## Figures and Tables

**Table 1:** Features of clinically available antimicrobial coating. FRI: fracture related infection; OSE: one stage exchange; TSE: two stage exchange; PJI: periprosthetic joint infection.

Type of Antimicrobial Coating	DAC (Hydrogel)	ETN PROtect (Coating)
**Type of implants on which is applicable**	Plates Screws Nails Uncemented prosthesis	Only available for Expert Tibial Nail (De Puy Synthes companies)
**Mode of application**	Directly in the operating room	Applied to tibial nail during production
**Type of antimicrobials available**	Antibiotics are added, upon surgeon’s responsibility, by a list of mixable antibiotics	Gentamicin
**Intended use by manufacturer**	Prevention of infection in fractures and joint replacement	Prevention of infection in fractures
**Described use by literature**	Prevention of infection in fractures and joints replacement Reconstruction surgery for FRI OSE/TSE with cementless implant for PJI	Prevention of infection in fractures Reconstruction surgery for FRI
